# The effectiveness of value-based messages to engage gun owners on firearm policies: a three-stage nested study

**DOI:** 10.1186/s40621-022-00394-6

**Published:** 2022-10-03

**Authors:** Claire Boine, Michael Siegel, Abdine Maiga

**Affiliations:** 1 Department of Community Health Sciences, Boston University School of Public Health, 801 Massachusetts Avenue, Boston, MA 02118 USA; 2 Department of Public Health and Community Medicine, Tufts University School of Medicine, Boston, MA USA; 3 Centre d’Etudes, de Recherches, de Communication et d’Animation Pour le Développement (CERCAD), Paris, France

**Keywords:** Gun violence prevention, Gun owners, Moral foundation questionnaire, Value-based messages, Health communication

## Abstract

**Background:**

Although gun owners overwhelmingly support violence prevention policies, they are hesitant to speak up publicly to advocate for these policies. We tested a series of communication messages on gun owners’ level of support for various firearm violence prevention policies and on their willingness to engage in gun violence prevention advocacy.

**Methods:**

We conducted three consecutive experiments, testing a total of thirteen messages on a sample of gun owners over 18 years old who live in the U.S. The first was a random experiment, the second a quasi-experiment, and the third a randomized control trial. The goal of having these varied methods was to develop messages applicable to different contexts with different levels of information about the audience.

**Results:**

The most effective message was a script showing respect for gun owners’ decisions to purchase a firearm and proposing a balanced policy roadmap to end gun violence, which led to an increase in gun owner’s willingness to engage in eight different advocacy activities. We also found a value-based message conveying loyalty to increase support for domestic violence related prohibitions and willingness to engage in advocacy for gun violence prevention policies.

**Conclusions:**

Public health professionals need to develop communication strategies that are aligned with gun owners’ values and that affirm respect for gun culture and for gun owners’ decisions to own a gun.

**Supplementary Information:**

The online version contains supplementary material available at 10.1186/s40621-022-00394-6.

## Introduction

Although gun owners overwhelmingly support policies such as universal background checks (Barry et al. 2019), firearm violence prevention is still wrongly perceived as a controversial field. The reason for this misconception is that perceived public opinion does not reflect actual public opinion on firearm violence prevention. It has been shown that the majority of gun owners are reluctant to publicly express their views, while a minority who opposes any legislation is very outspoken (Siegel and Boine 2020). There are multiple reasons for this unwillingness to speak up. First, gun owners who personally support firearm violence prevention policies tend to underestimate the proportion of other gun owners who share their views (Dixon et al. 2020). Second, many gun owners are reluctant to let others know that they own guns (Wallace 2017). Third, a majority of gun owners who support gun violence prevention policies are hesitant to speak up because they feel alienated and blamed for gun violence (Siegel and Boine 2020). As a result, those firearm owners who oppose firearm violence prevention are disproportionately heard. A key to moving the firearm debate forward would be to convince gun owners who support violence prevention policies to publicly express their support for these policies. This would prevent policymakers and the public from assuming that opponents of gun violence prevention, who are the most vocal, are representative of gun owners. It would in turn reduce the gap between actual and perceived public opinions on gun violence prevention, which would motivate policymakers to act since policymakers have been shown to be influenced by public opinion (Burstein 2003).

**Fig. 1:**
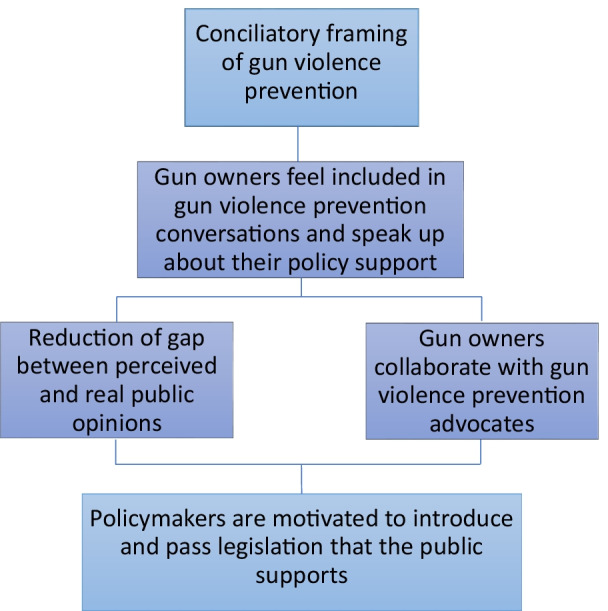
Theory of change. Presents the theory of change motivating this study

One challenge facing public health professionals is how to communicate effectively with gun owners to engage them in at least some level of gun violence prevention advocacy. Research has already shown that the opinions of gun owners on public policy can vary by race and ethnicity (Crifasi et al. 2021); now, we examine some of the ways language and framing may influence these preferences within and across groups. Previous research has demonstrated that words matter and that certain terms—such as gun control—may elicit opposition to firearm violence prevention policies (Betz et al. 2021). In this study, an experimental design tested the effectiveness of several messages conveying different values and intended to enhance existing support for firearm policies and to motivate gun owners to be willing to engage in gun violence prevention activity. Figure 1 represents this study’s theory of change.

Core values can be regarded as forming one of the most fundamental components of a group’s culture, representing the heart of the ideological system, and acting as identifying values which are symbolic of the group and its membership (Smolicz 1981). Sociologists have proposed different sets of core values over time. Recently, Jesse Graham, Jonathan Haidt, and their colleagues have developed the Moral Foundation Theory in which they identify seven fundamental values: care (an ability to feel and dislike the pain of others), reciprocity (proportionality/fairness), loyalty (self-sacrifice for the group), authority (deference to authority perceived as legitimate and respect for traditions), purity (disgust for what is perceived as unnatural or desecrated), autonomy (freedom), and equality (equal division of resources) (Graham et al. 2011; Haidt 2013).

Research has shown that the underlying core values people hold often predict their political ideology and affiliation, level of support for different policies, and willingness to engage on these issues (Bowe and Hoewe 2016; Dickinson et al. 2016; Graham et al. 2011; Haidt and Graham 2007; Silver 2017). For instance, Burton showed that the value of care was correlated with policy preferences on firearms (Burton et al. 2021). Similarly, a lack of consistency in values across groups with similar vested interests can overwhelm these similarities and lead to potential allies feeling removed or alienated from each other. In fact, even perceived threats to one’s values, if they are intertwined with individual or group identity, can be enough to mobilize resistance (Melzer 2009).

Core values can be used in crafting effective public health messages. In fact, it was demonstrated that most often the public does not value health in itself but value it for the autonomy and freedom it enables (Resnick and Siegel 2012). Freedom and autonomy are more fundamental core values than health. Therefore, when a debate has two sides, such as the one between public health professionals and the tobacco industry, and one side claims the value of health while the other focuses on individual freedom, the latter usually wins. An effective public health tool is thus to reframe an issue using the core values held by the audience we want to reach.

A frame is a way of packaging and positioning an issue to convey a certain meaning (Chapman and Lupton 1994; Rein and Schön 1996; Resnick and Siegel 2012; Ryan 1999; Wallack et al. 1993). In developing frames, public health practitioners must identify how to package an issue in a way that (1) presents a coherent core position; (2) evokes desired visual images; (3) uses recognizable catch-phrases; (4) suggests appropriate metaphors; (5) attributes responsibility for the problem to society; and (6) implies as a solution the policy being marketed by the practitioner (Resnick and Siegel 2012).

Framing has already had a demonstrable effect on the policy debate. The National Rifle Association (NRA) reframed gun culture in the late 1970s to attract a more conservative and ideologically extreme membership, showing the effectiveness of framing on gun policy (Melzer 2009). The reframing of mass shootings within the United States as a mental health crisis rather than isolated incidents has shifted public opinion towards more comprehensive prevention measures (DeFoster and Swalve 2017). Using this approach, the study team used a longitudinal survey to test the effectiveness of different value-based frames on: (1) support for different firearm policies; and (2) willingness to take different actions to engage on firearm policy.

## Methods

### Study design and data collection

At baseline, a nationally representative internet-based survey was conducted with 2086 gun owners using the Ipsos© KnowledgePanel® (KP), which is the largest national internet panel whose members were selected using representative sampling techniques. Respondents were eligible for inclusion in the study if they were 18 years of age or older, lived in the United States, and owned a firearm; efforts were also taken to ensure the sample was geographically representative of the national population. The respondents were asked questions about their firearm-related activities and preferences, their values, their opinions on gun policies, and their willingness to engage in a range of firearm violence prevention efforts. For the purposes of this study, firearm violence refers to homicide and interpersonal violence. Suicide prevention, also critical to reducing gun deaths, may be more effectively addressed by other suites of policies and mental health interventions. In a follow-up survey, we attempted to re-contact all 2086 respondents from the baseline survey. They received an email inviting them to take part in the follow-up survey. A total of 1550 respondents completed the follow-up survey, for a follow-up response rate of 74.3%. The differences in demographics between the baseline and the follow-up surveys are presented in Table 1.

**Table 1:** Comparison of key demographics between initial and follow-up survey respondents

	Original survey	Follow-up survey
Number of participants	2086	1550
Average age (rounded)	55.9 (56)	56.6 (57)*
White, non-Hispanic	1740 (76.3%)	1300 (83.9%)
Black, non-Hispanic	136 (8.2%)	100 (6.5%)
Other, non-Hispanic	42 (4.1%)	29 (1.9%)
Hispanic	113 (10.1%)	81 (5.2%)
2 + races, non-Hispanic	55 (1.4%)	40 (2.6%)
Male respondents	1518 (68.7%)	1161 (74.9%)
Identifies as Republican	1107 (51.4%)	840 (54.2%)
Identifies as Democrat	522 (25.0%)	389 (25.1%)

*Follow-up survey was conducted 1 year later

**Fig. 2:**
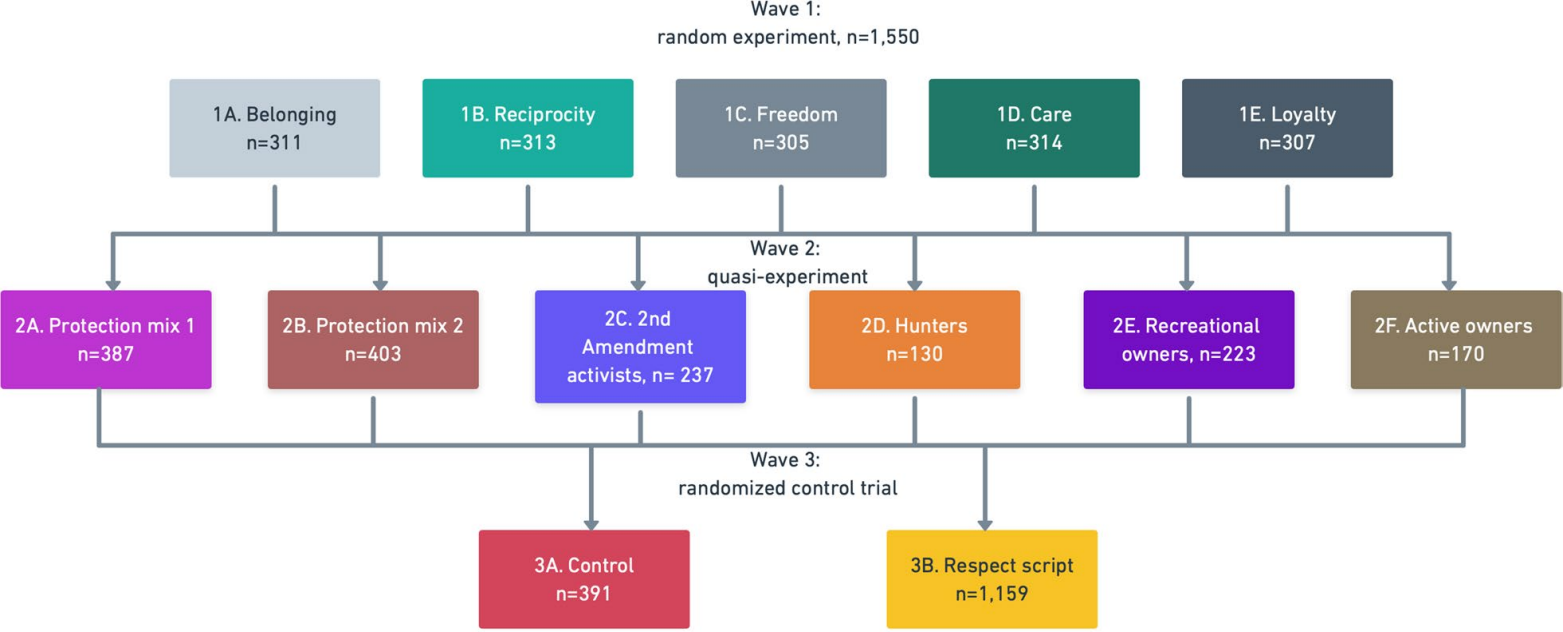
Study design. Participants were presented with three consecutive experiments in wave 1, wave 2, and wave 3

The follow-up survey contained a nested model of three consecutive interventions: a random experiment, a quasi-experiment, and a randomized control trial. These were meant to test the effectiveness of different messages. The study design is summarized in Fig. 2.

Different consecutive experimental designs were conducted to test multiple types of hypotheses and maximize the usefulness of this study for practitioners and public health communicators. In the first experiment, the goal was to understand which values are the most effective in communicating with gun owners in general. The second experiment assesses the effectiveness of different messages based on gun owner’s profiles. Therefore, the conclusions drawn from this quasi-experiment can only be useful in contexts where practitioners target a specific subset of gun owners. In the third experiment a randomized control trial tests if a respect-based message is more effective than a neutral message when presenting statistics on firearm-related deaths. All the messages presented in the experiment can be found in Additional file 1: Appendix 1.

### Messages

The members of our research team developed three types of messages, using themes and language they heard in gun owner testimonies online and in conversations with gun owners. In experiments 1 and 2, messages followed the Narrative Policy Framework, which suggests that all policy narratives have generalizable structural elements such as a setting, characters (such as heroes, villains, and victims), a plot, and a moral of the story (Jones and Shanahan 2014; Schlaufer et al. 2022). They were written from the perspective of a gun owner who faced a challenge and presented a firearm violence prevention policy as a solution. The study was initially planned to include focus groups to test the messages before the follow-up survey, but due to delays related to the COVID-19 pandemic, this was not possible; focus groups were instead conducted after the survey and will be presented in a subsequent paper. In experiment 1, each message was meant to appeal to different moral values. In experiment 2, the messages were created in an attempt to reach each of six gun owner audience segments that emerged from a latent class analysis. We looked at the main characteristics of each identified latent group and tried to adapt each message to their profile. In the third experiment, the control message was a fact-based account of firearm violence statistics in the U.S. The treatment message focused on conveying respect for gun ownership and gun culture and was prompted by previous research showing that the reason why “gun owners do not publicly support gun violence prevention policies that they favor is a feeling of alienation because of their perception that gun control advocates do not respect gun culture generally and their decision to own a gun for self-defense specifically” (Siegel and Boine 2020). The messages were delivered in written form during the follow-up survey.

### Measures

In the follow-up survey, the respondents were asked a series of six questions assessing their support for various firearm-related policies (policy questions) as well as nine questions about their willingness to engage in gun violence prevention activities (engagement questions). They are all presented in Additional file 3: Appendix 3.

The policy questions assessed respondents’ support for: (1) prohibiting a person subject to a temporary domestic violence restraining order from having a gun for the duration of the order (domestic violence prohibitions); (2) background checks for private sales and at gun shows (universal background checks); (3) prohibiting gun possession by people deemed to be a risk to themselves or others (*risk prohibitions*); (4) giving law enforcement officers discretion in whether or not to approve a concealed carry permit application (*may issue laws*); (5) prohibiting a person convicted of a violent crime from having a gun (*violent crime*); (6) allowing people to shoot as a first option if threatened, without any duty to retreat (*stand your ground laws*). These policies were chosen because of their effectiveness in homicide prevention, which is the focus of this study. Universal background checks, may issue laws, and prohibitions against gun ownership by people convicted of violent misdemeanors in particular have been shown to be highly effective interventions against gun-related interpersonal violence (Humphreys et al. 2017; Kalesan et al. 2016; Siegel et al. 2019).

The engagement questions tested respondents’ willingness to engage in different activities in favor of firearm violence prevention: (1) willingness to contact a public official; (2) making a donation; (3) talking to friends or family members; (4) attending a public health advocacy meeting; (5) testifying at a public hearing; (6) writing a letter to the editor; (7) writing a comment in an internet discussion; (8) talking to other gun owners; (9) talking to non-gun owners. These questions are consistent with the relevant scholarship. Middlewood used contacting officials about gun policy, donating money to gun policy organizations, and discussing firearms on the internet as measures of engagement (Middlewood 2021). Schwartz asked gun owners twelve questions to assess their level of political engagement, including whether they contacted a public official, donated money to a gun rights group, attended a public meeting, talked to friends or family members about firearm policy, or wrote to a newspaper (Schwartz 2021). Each respondent was assigned an *engagement score* by averaging out their responses to the nine questions.

### Intervention

For each experiment, the main goal was to assess the effectiveness of the set of messages of this experiment. Therefore, the response variable was the score right after this experiment minus the score just before the experiment.

#### First experiment

The participants were randomly assigned to one of five different messages on keeping firearms away from domestic abusers. These messages were developed to each represent one or two main values: belonging, reciprocity, freedom, care, and loyalty. These values were chosen because they have been shown to be instrumental to policy preferences (Haidt 2013; Resnick and Siegel 2012). The six policy questions and nine engagement questions were then repeated for each respondent to compare the responses before and after exposure to the message.

#### Second experiment

The second intervention was a quasi-experiment. The study team first used latent class analysis to create clusters of participants based on their primary reasons for owning firearms and their participation in various gun-related activities. We used both the Akaike Information Criterion (Akaike 1973) and the Bayesian Information Criterion (Schwarz 1978) to determine the number of classes to retain and found six groups. The results of the latent class analysis are shown in Table 2.

**Table 2:** Six-class model: probability of endorsing item given latent class used in Experiment 2

	First class	Second class	Third class	Fourth class	Fifth class	Sixth class
Class probability	25%	26%	15%	8%	14%	11%
Number of respondents	387	403	237	130	223	170
*Gender*						
Male	65%	77%	81%	82%	75%	78%
Female	35%	23%	19%	18%	25%	22%
*Race*						
White, non-Hispanic	80%	78%	84%	96%	87%	90%
Black, non-Hispanic	11%	9%	4%	2%	2%	4%
Other, non-Hispanic	2%	2%	2%	0%	2%	2%
Hispanic	5%	8%	5%	2%	5%	2%
2 + races, non-Hispanic	2%	3%	5%	0%	3%	2%
*Reported political ideology*						
Conservative	47%	55%	68%	48%	39%	57%
Moderate	32%	33%	26%	28%	32%	27%
Liberal	21%	12%	7%	24%	29%	16%
*Primary reason for owning a firearm*						
Self-protection	27%	21%	7%	0%	0%	11%
Family protection	58%	64%	54%	1%	0%	17%
Community protection	0%	0%	0%	0%	0%	0%
Exercising one’s Second Amendment	6%	6%	16%	1%	3%	10%
Collecting	0%	1%	4%	0%	16%	6%
Family Tradition	0%	0%	0%	2%	31%	2%
Hunting	1%	0%	11%	85%	0%	27%
Target shooting	0%	3%	4%	2%	38%	20%
*Gun related activities*						
Going to a shooting range at least once a month	1%	22%	31%	0%	3%	18%
Carried favorite firearm in the past month	11%	42%	62%	26%	0%	41%
Subscribes to a gun-related magazine	3%	18%	44%	3%	3%	29%
*NRA membership*						
Membership in the NRA	2%	12%	39%	2%	2%	25%
*Identity*						
Owning a gun is a significant aspect of own identity	9%	15%	43%	5%	4%	13%
*Number of firearms owned*						
Total number of firearms owned	2.7	3.8	10.5	3.8	3.3	9.4
*Moral foundation value score*						
Care	3.3	3.2	3.0	3.1	3.3	2.9
Reciprocity	3.1	3.3	3.7	3.1	3.1	3.2
Loyalty	2.3	2.5	2.8	2.3	2.1	2.5
Authority	3.3	3.3	3.5	3.2	3.0	3.2
Purity	2.7	2.7	2.8	2.6	2.4	2.6
Autonomy	3.1	3.3	3.8	3.1	3	3.4
Equality	2.3	2.2	2.1	2.4	2.2	2.1

The first group is mostly composed of people who have a firearm either to protect their family (58%) or to protect themselves (27%). It has the highest proportion of women (35%) and of non-white people (20%). It has a relatively high proportion of liberals (20%) and a low rate of NRA membership (2%). Only 1% of them go to the shooting range monthly and 11% have carried their favorite firearm in the past month. The second group is also composed of people who have a firearm either to protect their family (64%) or to protect themselves (21%) and is 77% men. It is slightly more conservative (55%) and has a higher NRA membership rate than the first class (12%). The main difference with the first group is that they are using their firearms. Forty-two percent of them have carried their favorite firearm in the past month, 22% go to the range monthly, and 18% are subscribed to gun-related magazines. The third group contains people who own on average 10 firearms. They use them for multiple purposes, but mainly to protect their family (54%), to exercise the Second Amendment (16%), and for hunting (11%). This group has the highest proportion of NRA members (39%) and of people who think that gun ownership is a core part of their identity (43%). They strongly value autonomy and reciprocity. The fourth group is composed of hunters (85%) and owners who need to manage pests on their property (9%). The fifth group consists of more liberal gun owners who happen to have a gun either for target shooting (38%), collecting (16%), managing pests (11%), or out of family tradition (31%). They may have received their firearm as a gift or may have inherited it. They have the lowest proportion of people who view gun ownership as an identity (4%) and the lowest proportion of NRA members (2%). We call them “coincidental” gun owners. The sixth group is a mix of hunters (27%), target shooters (20%), family protectors (17%), self-protectors (11%), Second Amendment advocates (10%) and collectors (6%). This group has a high proportion of NRA members (25%). We will call this group active owners because they are involved in multiple gun-related activities, as well as Second Amendment advocacy. After conducting the latent class analysis, we developed a segment-specific message for each group. The messages discussed the importance of universal background checks. As in the first experiment, the six policy questions and nine engagement questions were then repeated to compare the responses before and after exposure for each respondent.

#### Third experiment

The third experiment is a randomized control trial with a treatment message delivered to 75% of our sample while the other 25% is exposed to a control message. The treatment message (respect script) addresses the respondents directly and presents them with a roadmap to bring gun owners and non-gun owners together to agree on a set of policies. It focuses on respecting the decision of gun owners to arm themselves. The control message is a short paragraph with detailed data and statistics on firearm violence prevention in the U.S. This time, after exposure to the control or treatment message, each respondent was asked only the engagement questions again, and the groups’ results were compared.

### Hypotheses

#### Hypothesis 1

In experiments 1 and 2, for each experiment, the level of support for the policy described in the message should increase after exposure to the message.

#### Hypothesis 2

In experiments 1 and 2, the engagement score should increase after exposure to the message.

#### Hypothesis 3

In experiment 3, the change in engagement score for the treatment group should be higher than the change in engagement score for the control group after the intervention.

### Data analysis

For the data analysis, we used R, and especially the lmerTest package. Our goal was to measure the effectiveness of each of the six messages with repeated measurements. We analyzed the results of the first experiment using a linear regression with, as the dependent variable, the change between the baseline responses and the responses after the first experiment.

Because respondents within each group in the second experiment had been exposed to different previous messages, and because the effect measured after the second experiment resulted from exposure to both the first and the second, experiments 1 and 2 are nested. The intraclass correlation shows how much, on average, the response variable varies from one experiment group to the other inside of experiment 2. In this case, those correlations were less than 1%, which means that experiment 1 does not have that much of an impact on experiment 2. The study group analyzed the results of experiment 2 using a linear mixed effect model with a random effect for experiment 1 and a fixed effect for experiment 2. The dependent variable was thus the difference between the responses after the first and second experiments and each respondent is compared against themselves. This model allows us to single out the effect of the second experiment while controlling for all the combinations of messages between the first and the second experiments.

For the third experiment, to examine the difference in effectiveness between the control group and the treatment group (exposed to the respect script), we compare the groups against each other as opposed to against themselves over time. Two random effects are introduced to account for the effect of experiment 1 and experiment 2 on the model. For both random variables, the intraclass correlation is less than 1% so the effects were negligible.

## Results

The full results from all three experiments are presented in Additional file 2: Appendix 2. Tables 3, 4, and 5, respectively, show the statistically significant results from experiments 1, 2, and 3.

**Table 3:** Abbreviated results from the first experiment

Dependent variable	Independent variable	Coefficient	Stand. Er	Pr( >|t|)
Universal background checks	1B. Reciprocity	− 0.07	0.03	0.03
Universal background checks	1C. Freedom	− 0.06	0.03	0.04
Domestic violence prohibitions	1B. Reciprocity	0.09	0.03	0.01
Domestic violence prohibitions	1E. Loyalty	0.13	0.03	0.00
Risk prohibitions	1B. Reciprocity	0.07	0.03	0.03
Risk prohibitions	1E. Loyalty	0.11	0.04	0.00
May issue laws	1C. Freedom	0.07	0.03	0.04
Stand-your-ground	1B. Reciprocity	0.09	0.04	0.01
Engagement score	1A. Belonging	0.17	0.07	0.02
Engagement score	1E. Loyalty	0.33	0.07	0.00

**Table 4:** Abbreviated results from the second experiment

Dependent variable	Independent variable	Coefficient	Stand. Er	Pr( >|t|)
Domestic violence prohibitions	2E. Recreational owners	0.06	0.03	0.04
Risk prohibitions	2C. 2nd amendment activists	0.09	0.03	0.01
Risk prohibitions	2E. Recreational owners	0.08	0.03	0.02
Engagement score	2E. Recreational owners	−0.15	0.07	0.04

**Table 5:** Abbreviated results from the third experiment

Dependent variable	Independent variable	Coefficient	Stand. Er	Pr( >|t|)
Contacting a public official	Control	− 0.07	0.10	0.48
Contacting a public official	Respect script	0.36	0.11	0.00
Talking to friends or family about prevention	Control	− 0.18	0.10	0.08
Talking to friends or family about prevention		0.35	0.11	0.00
Attending a meeting of public health advocates	Control	0.00	0.09	1.00
Attending a meeting of public health advocates	Respect script	0.32	0.10	0.00
Testifying at a public hearing in favor of a policy	Control	− 0.01	0.08	0.95
Testifying at a public hearing in favor of a policy	Respect script	0.26	0.10	0.01
Writing a letter to the editor in favor of a policy	Control	0.01	0.07	0.88
Writing a letter to the editor in favor of a policy	Respect script	0.18	0.08	0.03
Writing a comment online in favor of a policy	Control	− 0.02	0.08	0.78
Writing a comment online in favor of a policy	Respect script	0.23	0.09	0.01
Gaining support from other gun owners	Control	0.10	0.09	0.30
Gaining support from other gun owners		0.26	0.10	0.01
Gaining support from non-gun owners	Control	0.07	0.09	0.44
Gaining support from non-gun owners	Respect script	0.23	0.10	0.03
Engagement score (average)	Control	− 0.01	0.06	0.93
Engagement score (average)		0.26	0.07	0.00

### Experiment 1

Table 3 presents the abbreviated results of the first experiment, in which respondents were exposed to messages on domestic violence. Only the responses to the policy questions and the average engagement score are shown. The results for each of the nine engagement questions are presented in Additional file 4: Appendix 4. Exposure to message 1A (belonging) had no effect on policy support but was significantly associated with an increase in the engagement score. Exposure to message 1B (reciprocity) was significantly associated with an increase in support for domestic violence prohibitions, risk prohibitions, and stand-your-ground laws, and with a decrease in support for universal background checks. Exposure to message 1C (freedom) was associated with a decrease in support for universal background checks and an increase in support for may issue laws. Exposure to message 1D (care) has no effect on policy support or willingness to engage in violence prevention. Exposure to message 1E (loyalty) was associated with an increase in support for domestic violence prohibitions and risk prohibitions, in addition to an increase in willingness to engage in firearm violence prevention activities.

### Experiment 2

Table 4 presents the results of the second experiment, in which respondents were exposed to messages on universal background check laws. Only the responses to the policy questions and the average engagement score are shown. The results for each of the nine engagement questions are presented in Additional file 4: Appendix 4. None of the messages had any impact on support for universal background checks. Exposure to the messages shown to groups 1, 2, 4, and 6 had no effect on policy support or willingness to engage. Exposure to message 2C, targeting the gun rights advocates, was positively associated with an increase in support for policies preventing people who are a risk to themselves or society from having a gun. Exposure to message 2E, targeting coincidental firearm owners, such as the majority of group 4, was associated with an increase in support for domestic violence prohibitions as well as prohibitions aimed at people who are a risk to themselves or others. However, it was also correlated with a decrease in willingness to engage in gun violence prevention activities.

### Experiment 3

Table 5 presents the results of the third experiment. The respect script is significantly associated with an increase in willingness to engage in all possible activities except for one (donating money to a gun violence prevention organization). It is also associated with an increase in the average engagement score.

## Discussion

### Experiment 1

Of all the messages, the most effective one was message 1E on loyalty, which was associated with an increase in support for domestic violence prohibitions, the policy the message was about. It also led respondents to be more willing to engage in firearm violence prevention activities. Message 1E starts with a narrative from a man who recalls hunting with his father. It focuses on loyalty to the father (“every time I go out hunting, I think of him”) and respect (“respect for the animal, respect for the process, and respect for the firearm”). It goes on to describe that it is wrong for some men to kill their wives and it advocates for removing firearms from the hands of domestic abusers. It closes on a value statement appealing to responsibility (“making sure that every gun owner is a responsible gun owner”).

Message 1B, which appeals to responsibility, had mixed effects and was thus counterproductive. On the one hand, it was significantly associated with an increase in support for domestic violence prohibitions and risk prohibitions. On the other hand, it was associated with an increase in support for stand-your-ground laws, and with a decrease in support for universal background checks. It is counterproductive in that stand-your-ground laws and the absence of universal background checks are correlated with an increase in crime (Humphreys et al. 2017; Kalesan et al. 2016; McClellan and Tekin 2017; Siegel et al. 2019). The message was meant to first reaffirm respondents’ values before exposing them to new information. Research has shown that a common myth is that women are assaulted by strangers. This “stranger danger” has been operationalized by the firearm lobby to sell more guns to women (Carlson 2014). In reality, 90.4% of women in the U.S. who are murdered are killed by someone they know, including 44% by a domestic partner (Fridel and Fox 2019). The first part of the message therefore built on respondents’ prior assumptions (“women getting assaulted and killed by strangers”) before informing them that “40% of women who get killed in the U.S. are murdered by their partner.” The call to action was “keep guns out of the hands of domestic abusers.” In further statistical analysis, we looked to see if the increase in support for the prohibitions, and the decrease in support for universal background checks combined with the increase in support for stand-your-ground laws happened in the same or different respondents. In those exposed to 1B, there was almost no overlap between those two subgroups. A possible interpretation is that some respondents may remember the domestic violence component of the message, while some may only recall the part about arming oneself in self-defense against strangers. This could come from a selection bias according to which some respondents only remember the information that matches their prior knowledge.

Message 1A, based on the core value of belonging, had no effect on policy support but was significantly associated with an increase in the engagement score. Message 1A focuses on lobbying and advocacy. It is the story of a gun owner who has firearms to protect their family but doesn’t identify with gun owners on TV. They become upset when they find out that the NRA lobbies against laws to prevent domestic abusers from acquiring guns, and they join a gun violence prevention advocacy group. It is possible that it increases the engagement score because it disproportionately focuses on civic engagement and that the policy component of the message is not as highlighted. It is also possible that the mention of the NRA was counterproductive. Narrative Policy Framework teaches us that stories are more effective when focusing on a hero instead of a villain and framed in positive terms (Jones and Shanahan 2014).

Exposure to message 1C (freedom) was counterproductive since it is associated with a decrease in support for universal background checks and an increase in support for may issue laws. Message 1C starts by reaffirming the reader’s belief that freedom is at the basis of American culture (“that’s why I believe in the Second Amendment”). Then, it reframes freedom in terms of “freedom from getting killed in their own house.” It is possible that mentioning the Second Amendment at the beginning of the message activates opposition to universal background checks. It is also possible that it activates deference to authority or the police, which would explain why it correlates with an increase in support for may issue laws.

Experiment 1 carries some limitations. The messages displayed were not perfectly analogous. Although the survey responses of participants in the first experiment were compared against themselves and there was no direct between-group comparison, having messages more similar to one another would have made the interpretation of the results easier. For instance, message 1A introduces some negative language about the NRA, and has a smaller policy component. Message 1B is the only message specifically establishing the gender of the narrator. These differences introduce more complexity in the interpretation of results.

### Experiment 2

Most messages from experiment 2 were ineffective, except for messages 2C and 2E which had some limited effects.

Message 2C starts by reaffirming the reader’s values and beliefs and asserts that “the Second Amendment right to own firearms is one of the most important freedoms that Americans enjoy.” Whereas a similar message was shown to a randomized group in the first experiment, this time, the script targets a group of gun owners engaged in Second Amendment advocacy. It goes on to say that irresponsible gun use tarnishes the reputation of law-abiding citizens. It ends on a call to action: “we hope that you will be willing to work with us to promote universal background checks at the federal level to make sure that every gun owner is a responsible gun owner.” Interestingly, it has no effect on support for universal background checks but it increases support for policies preventing people who are a risk to themselves or society from having a gun. Given that the target group consists of gun owners who are already engaged in Second Amendment advocacy and are probably sensitive to messages from the gun lobby, it is possible that their position is firm on policies that are named (“universal background checks,” “may issue laws”…) that they may have heard a lot about already. However, it is possible that they support the content of the same policies when phrased in terms of principles (“prohibiting gun possession by people deemed to be a risk to themselves or others”). Whereas the latter was framed as a principle in the survey, universal background checks were named as such.

Message 2E targets recreational gun owners and focuses on people and children dying because guns get into the wrong hands. Exposure to message 2E, targeting coincidental firearm owners, was associated with an increase in support for domestic violence prohibitions as well as prohibitions aimed at people who are a risk to themselves or others. However, it was also correlated with a decrease in willingness to engage in gun violence prevention activities. It is difficult to know why this message has such contradictory effects. It is possible that gun owners do not want to talk about children dying from gun violence to other people. It is also possible that this message activated coincidental gun owners’ fear of telling others that they own a gun.

### Experiment 3

Experiment 3 features a control message focusing on dispassionate statistics, and a treatment message written from the perspective of public health professionals. It starts by showing respect to gun owners for their decision to own a firearm. It then proposes a conversation on ways to reduce gun violence. It suggests a specific roadmap that would take any proposal to ban guns or any specific types of guns off the table and focus on making sure that people who pose a high risk of violence based on their criminal history should not be allowed to possess guns. The results of experiment 3 are very consistent. The respect script is significantly associated with an increase in willingness to engage in all possible gun violence prevention activities except for donating money to a gun violence prevention organization. This exception may be due to preexisting suspicion toward existing gun violence prevention. The results of experiment 3 are consistent with the literature showing that the use of emotions in political communication is significantly more effective than dispassionate statistics (Westen 2007). They also validate the hypothesis that even though “on a population basis, a higher prevalence of household gun ownership is associated with higher rates of firearm-related death, public health professionals must still respect the decision that gun owners have made to protect themselves and their families. Educating gun owners on the risks associated with gun ownership is important but needs to be done without criticizing gun owners or attacking gun culture” (Siegel and Boine 2020). This finding is consistent with the findings from the loyalty message, which contains the word “respect” four times. It is possible that gun owners value respect more than the general population. It seems that a good public health communication strategy could undo years of heated public debate that led gun owners to feel alienated and disrespected by public health professionals (Siegel and Boine 2020).

## Policy implications

The main policy implication is that communication with firearm owners must consist of first expressing respect for their decision to own a gun, which should improve their feeling of alienation and build a bridge, allaying perceived animosity. This is consistent with Intergroup Emotions Theory. For instance, Maitner et al. (2016) write that “if individuals can shift the dynamic between ingroup and target outgroup, reducing the threat perceived in the outgroup by bringing it into the ingroup or perceiving some overlapping shared category, animosity will be reduced.”

Another implication for public health campaigns aimed at reducing gun violence is that the value that is most effective in mobilizing a random subset of gun owners is loyalty. It was shown that most gun owners become interested in firearms because of the social bonds they associate them with. For instance, many gun owners become interested in firearms either during childhood or early adulthood and firearms evoke images such as going hunting with a grandparent or going to the range with a romantic partner (Harel et al. 2021). If firearms are associated with someone’s close relationships, a message evoking these bonds and implicitly conveying the value of loyalty seems to be very effective.

## Limitations

The first limitation of this study is that the sample size of certain groups in the second experiment, due to the latent class analysis, made it impossible to reach statistical significance in some cases where there could have been interesting patterns.

Another limitation is that the policy measures at the end of experiment 3 could not be repeated, due to survey duration constraints.

Finally, the engagement questions did not include whether the respondents were single-issue voters based on firearm policy, even though gun ownership status is one of the strongest predictors of policy preference (Aronow and Miller 2016; Joslyn 2020; Joslyn et al. 2017; Lacombe 2019).

## Conclusion

Among the thirteen messages tested in total, only a few were effective. This finding shows that using classic Second Amendment rhetoric for the purpose of gun violence prevention may be ineffective or even counterproductive when it activates gun owners in the opposite way. The most effective message was the one most likely to reflect actual messaging from public health professionals and showing respect toward gun owners to include them in the conversation. We conclude that public health professionals need to engage with firearm owners within their own ideological framework. By respecting the right to firearm ownership, misconceptions of animosity created by the firearm industry can be overcome to inform public perception and support for public policy.

## Supplementary Information

**Additional file 1**. **Appendix 1**: All messages presented to the respondents. This appendix presents all the messages that were crafted for the study and that some respondents have been exposed to.

**Additional file 2**. **Appendix 2**: Full results. This appendix shows the results of the statistical analyses to measure the effect of the messages on policy support and willingness to engage, regardless of whether statistical significance was reached. Experiment 1 was analyzed using a linear regression with, as the dependent variable, the change between the baseline responses and the responses after the first experiment. Experiment 2 was analyzed using a linear mixed effect model with a random effect for experiment 1 and a fixed effect for experiment 2. Experiment 3 was examined using a linear mixed effect model comparing the control and the intervention groups, with random effects for experiments 1 and 2. The different measures of engagement are combined into the engagement score.

**Additional file 3**. **Appendix 3**: Questions posed to the respondents. This appendix presents all the questions asked to the survey respondents. This appendix shows the results of the statistical analyses.

**Additional file 4**. **Appendix 4**: Results of the first and second experiment for each engagement question. This appendix shows the results of the statistical analyses to measure the effect of the messages in experiments 1 and 2 on respondents’ willingness to engage in gun violence prevention in each of nine particular ways.

## Data Availability

The datasets generated during the current study are available from the corresponding author on reasonable request.

## Abbreviation

NRA: National Rifle Association
