# Supplementary material for: The effectiveness of value-based messages to engage gun owners on firearm policies: a three-stage nested study
Source: Inj Epidemiol. 2022 Oct 3;9:30. doi: 10.1186/s40621-022-00394-6 (PMC9527730; doi:10.1186/s40621-022-00394-6)
Supplement: Supplementary file 1 — Additional file 1. Appendix 1: All messages presented to the respondents. This appendix presents all the messages that were crafted for the study and that some respondents have been exposed to. [file 40621_2022_394_MOESM1_ESM.docx]

Appendix 1. All messages presented to the respondents

**Message 1A. Belonging**

I don’t really think of myself as a gun owner. I bought a handgun to protect my family and my home, but I don’t ever use it. In fact, when I see gun owners interviewed on TV, I never identify with them. However, when I see angry gun violence prevention advocates who just want to take away all the guns, it makes me really mad. I think they mean well, but they don’t know anything about firearms. They should let me exercise my right to protect my loved ones. A few weeks ago, I heard that the NRA was lobbying against laws that would prevent domestic abusers from getting guns in my state. That’s what decided me to contact gun violence prevention advocates from my community. I wanted them to know that not all gun owners are like these guys on TV. I started teaching them about firearms and took them to the range, and I have helped them advocate to keep guns out of the hands of domestic abusers. That’s why I want you to join our effort to make sure that all gun owners are responsible gun owners.

**Message 1B. Reciprocity**

As women, we constantly fear things that men don’t have to think about. It’s like we live in a different world. When I turn on the news and hear about women getting assaulted and killed by strangers, it makes my blood boil. But we shouldn’t have to be victims. That’s why I have a gun and I am trained to use it. If robbers and assailants show up, I will be the first responder on the scene. And I want women to be safe from other threats as well. I know that 40% of women who get killed in the U.S. are murdered by their partner. That’s why I want you to join our effort to keep guns out of the hands of domestic abusers. Guns are equalizers, they are for good people to not be victimized, they're not for bullies. Let’s make sure that all gun owners are responsible gun owners.

**Message 1C. Freedom**

Freedom is at the basis of American culture. That’s why I believe in the Second Amendment. Everybody should have the right to protect themselves and their family. I carry a gun because I want to be free from criminals and I want to be safe inside my home. That’s also why I support laws that keep guns out of the hands of domestic abusers. Because everybody should be free from getting killed in their own house. I want you to join our effort to make sure that all gun owners are responsible gun owners.

**Message 1D. Care**

I have guns to protect my family. When I go to bed, I feel safe, because I know if an intruder comes into our home, I can protect my wife and children. However, I know that many people are not responsible like me and end up using their guns in the comfort of their home, against their own spouse. That’s why I support laws to keep guns out of the hands of domestic abusers. I want you to join our effort to make sure that all gun owners are responsible gun owners.

**Message 1E. Loyalty**

I grew up hunting with my dad. We’d wake up when it was pitch dark out, put our gear in the truck, and head out for the day. There was a rhythm to it. Even though my dad’s been gone for years now, every time I go out hunting, I think of him. He taught me so much - how to hunt, yes, but above all that, respect. Respect for the animal, respect for the process, and respect for the firearm. From day one, he made sure I knew absolutely everything I needed to handle that weapon safely, and that has made me a responsible gun owner. I had to prove to him that I could handle the responsibility and when I did, it opened up a whole new world to me. That’s why, when I hear about men using their guns to kill their wives, it makes me sick. It is wrong and it gives responsible gun owners like me a bad name. Guys who do that don’t get to use the Second Amendment to commit their crimes. That’s why I’ve called my legislators to let them know I support keeping guns out of the hands of domestic abusers. It is part of the process of making sure that every gun owner is a responsible gun owner.

**Message 2A. Protection mix 1**

As public health researchers, we respect your decision to have purchased a firearm for self-defense and acknowledge your right to protect yourself and your family. This is why we want to include you in our effort to combat irresponsible gun use. Too many people, including children, are dying because guns are getting into the wrong hands. We hope that you will be willing to work with us to promote universal background checks at the federal level to make sure that every gun owner is a responsible gun owner.

**Message 2B. Protection mix 2**

As public health researchers, we respect your decision to have purchased a firearm for self-defense and acknowledge your Second Amendment right to protect yourself and your family. We also respect gun culture and think that it is an important part of American culture. We know that most gun owners are law-abiding and get their firearms the right way by being willing to go through a background check, to weed out criminals who are trying to get their hands on a gun. It doesn’t seem fair that many criminals are able to get guns the easy way by avoiding background checks. We hope that you will be willing to work with us to promote universal background checks at the federal level to make sure that every gun owner is a responsible gun owner.

**Message 2C. Second Amendment activists**

As public health researchers, we recognize that the Second Amendment right to own firearms is one of the most important freedoms that Americans enjoy. For the past decade, gun crime has increased significantly in our country. These irresponsible criminals are tarnishing the reputation of law-abiding citizens. This is why we want to include you in our effort to combat irresponsible gun use. We oppose laws that would take any gun away from law-abiding citizens. We think a more effective approach is to try to keep the guns out of the hands of would-be criminals. The fact that criminals will always try to get their hands on a gun doesn’t mean we should make it easier for them. We hope that you will be willing to work with us to promote universal background checks at the federal level to make sure that every gun owner is a responsible gun owner.

**Message 2D. Hunters**

As public health researchers, we respect the tradition of hunting and other recreational uses of firearms, and we recognize their importance in American culture. This is why we want to include you in our effort to combat irresponsible gun use. Too many people, including children, are dying because guns are getting into the wrong hands. We hope that you will be willing to work with us to promote universal background checks at the federal level to make sure that every gun owner is a responsible gun owner.

**Message 2E. Recreational**

As public health researchers, we respect the tradition of target shooting and other recreational uses of firearms, and we recognize their importance in American culture. This is why we want to include you in our effort to combat irresponsible gun use. Too many people, including children, are dying because guns are getting into the wrong hands. We hope that you will be willing to work with us to promote universal background checks at the federal level to make sure that every gun owner is a responsible gun owner.

**Message 2F. Active owners**

As public health researchers, we recognize that the Second Amendment right to own firearms is one of the most important freedoms that Americans enjoy. We oppose laws that would take any gun away from law-abiding citizens. We think a more effective approach is to try to keep the guns out of the hands of would-be criminals. The fact that criminals will always try to get their hands on a gun doesn’t mean we should make it easier for them. This is why we hope that you will be willing to work with us to promote universal background checks at the federal level to make sure that every gun owner is a responsible gun owner.

**Message 3A. Informational script (control)**

In 2018, nearly 40,000 people died from firearm violence. About 14,000 of these victims died from gun homicides, and another 24,000 died from gun suicides. An additional 458 deaths occurred from accidental gun shootings. About 1.7 million people have died from firearms in the U.S. between 1968 and 2019. This number includes all deaths resulting from a firearm, including suicides, homicides, and accidents. Compared to 22 other high-income nations, the U.S. gun-related homicide rate is 25 times higher. Although it has half the population of the other 22 nations combined, among those 22 nations studied, the U.S. had 82 percent of gun deaths, 90 percent of all women killed with guns, 91 percent of children under 14 and 92 percent of young people between ages 15 and 24 killed with guns.

**Message 3B. Respect Script (treatment)**

Many gun owners feel that gun control advocates do not respect their decision to purchase a gun for self-defense. The researchers conducting this study believe that gun owners have the right to protect themselves and their families and we respect that decision. We respect gun culture and believe that gun owners know more than anyone else about responsible gun use. We therefore believe that gun owners need to be brought into the discussion about ways to reduce gun violence. We also believe that policies should be aimed not at regulating the types of guns that can be sold (e.g., assault weapon bans), but at making sure that people who pose a high risk for violence based on their criminal history should not be allowed to possess guns and stronger efforts should be made to enforce those restrictions. Our hope is that gun owners and non-gun owners can come together to design a set of gun violence prevention policies that all can agree on. That discussion would begin by taking any proposal to ban guns or any specific types of guns off the table. It would also begin by soliciting the opinions of gun owners about what they feel would be the most effective policies for reducing gun violence
