# Supplementary material for: The effectiveness of value-based messages to engage gun owners on firearm policies: a three-stage nested study
Source: Inj Epidemiol. 2022 Oct 3;9:30. doi: 10.1186/s40621-022-00394-6 (PMC9527730; doi:10.1186/s40621-022-00394-6)
Supplement: Supplementary file 2 — Additional file 2. Appendix 2: Full results. This appendix shows the results of the statistical analyses to measure the effect of the messages on policy support and willingness to engage, regardless of whether statistical significance was reached. Experiment 1 was analyzed using a linear regression with, as the dependent variable, the change between the baseline responses and the responses after the first experiment. Experiment 2 was analyzed using a linear mixed effect model with a random effect for experiment 1 and a fixed effect for experiment 2. Experiment 3 was examined using a linear mixed effect model comparing the control and the intervention groups, with random effects for experiments 1 and 2. The different measures of engagement are combined into the engagement score. [file 40621_2022_394_MOESM2_ESM.docx]

**Appendix 2. Full results**

| First experiment | | | | | | | | |
| --- | --- | --- | --- | --- | --- | --- | --- | --- |
| Dependent Variable | Independent Variable | | Coefficient | Stand. Er. | | | Pr(>\|t\|) | |
| Universal background checks | 1A. Belonging | | 0.02 | 0.03 | | | 0.46 | |
| Universal background checks | **1B. Reciprocity** | | **-0.07** | **0.03** | | | **0.03** | |
| Universal background checks | **1C. Freedom** | | **-0.06** | **0.03** | | | **0.04** | |
| Universal background checks | 1D. Care | | -0.03 | 0.03 | | | 0.40 | |
| Universal background checks | 1E. Loyalty | | 0.04 | 0.03 | | | 0.20 | |
| Domestic violence prohibitions | 1A. Belonging | | 0.01 | 0.03 | | | 0.70 | |
| Domestic violence prohibitions | **1B. Reciprocity** | | **0.09** | **0.03** | | | **0.01** | |
| Domestic violence prohibitions | 1C. Freedom | | 0.01 | 0.03 | | | 0.77 | |
| Domestic violence prohibitions | 1D. Care | | 0.05 | 0.03 | | | 0.12 | |
| Domestic violence prohibitions | **1E. Loyalty** | | **0.13** | **0.03** | | | **0.00** | |
| Risk prohibitions | 1A. Belonging | | 0.04 | 0.04 | | | 0.23 | |
| Risk prohibitions | **1B. Reciprocity** | | **0.07** | **0.03** | | | **0.03** | |
| Risk prohibitions | 1C. Freedom | | 0.01 | 0.04 | | | 0.71 | |
| Risk prohibitions | 1D. Care | | 0.01 | 0.03 | | | 0.78 | |
| Risk prohibitions | **1E. Loyalty** | | **0.11** | **0.04** | | | **0.00** | |
| May issue laws | 1A. Belonging | | -0.02 | 0.03 | | | 0.61 | |
| May issue laws | 1B. Reciprocity | | 0.06 | 0.03 | | | 0.08 | |
| May issue laws | **1C. Freedom** | | **0.07** | **0.03** | | | **0.04** | |
| May issue laws | 1D. Care | | 0.03 | 0.03 | | | 0.31 | |
| May issue laws | 1E. Loyalty | | 0.06 | 0.03 | | | 0.08 | |
| Violent crime prohibitions | 1A. Belonging | | 0.02 | 0.03 | | | 0.49 | |
| Violent crime prohibitions | 1B. Reciprocity | | -0.03 | 0.03 | | | 0.32 | |
| Violent crime prohibitions | 1C. Freedom | | -0.03 | 0.03 | | | 0.32 | |
| Violent crime prohibitions | 1D. Care | | -0.05 | 0.03 | | | 0.14 | |
| Violent crime prohibitions | 1E. Loyalty | | 0.02 | 0.03 | | | 0.55 | |
| Stand-your-ground | 1A. Belonging | | 0.02 | 0.04 | | | 0.54 | |
| Stand-your-ground | **1B. Reciprocity** | | **0.09** | **0.04** | | | **0.01** | |
| Stand-your-ground | 1C. Freedom | | 0.03 | 0.04 | | | 0.37 | |
| Stand-your-ground | 1D. Care | | 0.07 | 0.04 | | | 0.06 | |
| Stand-your-ground | 1E. Loyalty | | 0.00 | 0.04 | | | 0.93 | |
| Engagement score | **1A. Belonging** | | **0.17** | **0.07** | | | **0.02** | |
| Engagement score | 1B. Reciprocity | | 0.06 | 0.07 | | | 0.44 | |
| Engagement score | 1C. Freedom | | 0.09 | 0.07 | | | 0.24 | |
| Engagement score | 1D. Care | | 0.03 | 0.07 | | | 0.70 | |
| Engagement score | **1E. Loyalty** | | **0.33** | **0.07** | | | **0.00** | |
| Second experiment | | | | | | | | |
| Dependent Variable | Independent Variable | | Coefficient | Stand. Er. | | | Pr(>\|t\|) | |
| Universal background checks | 2A. Protection | | -0.01 | 0.03 | | | 0.59 | |
| Universal background checks | 2B. Protection | | 0.03 | 0.02 | | | 0.18 | |
| Universal background checks | 2C. 2nd Amendment activists | | -0.01 | 0.03 | | | 0.79 | |
| Universal background checks | 2D. Hunters | | -0.02 | 0.04 | | | 0.71 | |
| Universal background checks | 2E. Recreational owners | | 0.06 | 0.03 | | | 0.07 | |
| Universal background checks | 2F. Active owners | | 0.02 | 0.04 | | | 0.64 | |
| Domestic violence prohibitions | 2A. Protection | | -0.01 | 0.02 | | | 0.59 | |
| Domestic violence prohibitions | 2B. Protection | | 0.02 | 0.02 | | | 0.39 | |
| Domestic violence prohibitions | 2C. 2nd Amendment activists | | -0.05 | 0.03 | | | 0.13 | |
| Domestic violence prohibitions | 2D. Hunters | | -0.02 | 0.04 | | | 0.57 | |
| Domestic violence prohibitions | 2E. Recreational owners | | 0.06 | 0.03 | | | 0.04 | |
| Domestic violence prohibitions | 2F. Active owners | | 0.01 | 0.04 | | | 0.74 | |
| Risk prohibitions | 2A. Protection | | -0.01 | 0.03 | | | 0.68 | |
| Risk prohibitions | 2B. Protection | | 0.00 | 0.03 | | | 0.98 | |
| Risk prohibitions | 2C. 2nd Amendment activists | | 0.09 | 0.03 | | | 0.01 | |
| Risk prohibitions | 2D. Hunters | | -0.03 | 0.04 | | | 0.48 | |
| Risk prohibitions | 2E. Recreational owners | | 0.08 | 0.03 | | | 0.02 | |
| Risk prohibitions | 2F. Active owners | | 0.04 | 0.04 | | | 0.36 | |
| May issue laws | 2A. Protection | | 0.00 | 0.03 | | | 0.90 | |
| May issue laws | 2B. Protection | | 0.04 | 0.03 | | | 0.21 | |
| May issue laws | 2C. 2nd Amendment activists | | 0.06 | 0.04 | | | 0.12 | |
| May issue laws | 2D. Hunters | | 0.05 | 0.05 | | | 0.33 | |
| May issue laws | 2E. Recreational owners | | 0.03 | 0.04 | | | 0.51 | |
| May issue laws | 2F. Active owners | | 0.03 | 0.04 | | | 0.44 | |
| Violent crime prohibitions | 2A. Protection | | -0.03 | 0.03 | | | 0.21 | |
| Violent crime prohibitions | 2B. Protection | | -0.01 | 0.03 | | | 0.71 | |
| Violent crime prohibitions | 2C. 2nd Amendment activists | | -0.05 | 0.03 | | | 0.14 | |
| Violent crime prohibitions | 2D. Hunters | | -0.09 | 0.05 | | | 0.07 | |
| Violent crime prohibitions | 2E. Recreational owners | | -0.03 | 0.04 | | | 0.37 | |
| Violent crime prohibitions | 2F. Active owners | | -0.07 | 0.04 | | | 0.08 | |
| Stand-your-ground | 2A. Protection | | 0.00 | 0.03 | | | 0.93 | |
| Stand-your-ground | 2B. Protection | | 0.00 | 0.03 | | | 0.86 | |
| Stand-your-ground | 2C. 2nd Amendment activists | | 0.01 | 0.04 | | | 0.74 | |
| Stand-your-ground | 2D. Hunters | | -0.01 | 0.05 | | | 0.88 | |
| Stand-your-ground | 2E. Recreational owners | | -0.02 | 0.04 | | | 0.56 | |
| Stand-your-ground | 2F. Active owners | | 0.05 | 0.04 | | | 0.29 | |
| Engagement score | 2A. Protection | | -0.10 | 0.05 | | | 0.08 | |
| Engagement score | 2B. Protection | | -0.04 | 0.05 | | | 0.45 | |
| Engagement score | 2C. 2nd Amendment activists | | 0.10 | 0.07 | | | 0.15 | |
| Engagement score | 2D. Hunters | | 0.09 | 0.09 | | | 0.32 | |
| Engagement score | 2E. Recreational owners | | -0.15 | 0.07 | | | 0.04 | |
| Engagement score | 2F. Active owners | | 0.12 | 0.08 | | | 0.14 | |
| Third experiment | | | | | | | |  |
| Dependent Variable | | Independent Variable | Coefficient | | Stand. Er. | Pr(>\|t\|) | |  |
| Contacting a public official | | Control | -0.07 | | 0.10 | 0.48 | |  |
| Contacting a public official | | **Respect script** | **0.36** | | **0.11** | **0.00** | |  |
| Donating to a gun violence prevention organization | | Control | 0.05 | | 0.08 | 0.53 | |  |
| Donating to a gun violence prevention organization | | Respect script | 0.09 | | 0.09 | 0.31 | |  |
| Talking to friends or family about prevention | | Control | -0.18 | | 0.10 | 0.08 | |  |
| Talking to friends or family about prevention | | **Respect script** | **0.35** | | **0.11** | **0.00** | |  |
| Attending a meeting of public health advocates | | Control | 0.00 | | 0.09 | 1.00 | |  |
| Attending a meeting of public health advocates | | **Respect script** | **0.32** | | **0.10** | **0.00** | |  |
| Testifying at a public hearing in favor of a policy | | Control | -0.01 | | 0.08 | 0.95 | |  |
| Testifying at a public hearing in favor of a policy | | **Respect script** | **0.26** | | **0.10** | **0.01** | |  |
| Writing a letter to the editor in favor of a policy | | Control | 0.01 | | 0.07 | 0.88 | |  |
| Writing a letter to the editor in favor of a policy | | **Respect script** | **0.18** | | **0.08** | **0.03** | |  |
| Writing a comment online in favor of a policy | | Control | -0.02 | | 0.08 | 0.78 | |  |
| Writing a comment online in favor of a policy | | **Respect script** | **0.23** | | **0.09** | **0.01** | |  |
| Gaining support from other gun owners | | Control | 0.10 | | 0.09 | 0.30 | |  |
| Gaining support from other gun owners | | **Respect script** | **0.26** | | **0.10** | **0.01** | |  |
| Gaining support from non-gun owners | | Control | 0.07 | | 0.09 | 0.44 | |  |
| Gaining support from non-gun owners | | **Respect script** | **0.23** | | **0.10** | **0.03** | |  |
| Engagement score (average) | | Control | -0.01 | | 0.06 | 0.93 | |  |
| Engagement score (average) | | **Respect script** | **0.26** | | **0.07** | **0.00** | |  |
