# Supplementary material for: The effectiveness of value-based messages to engage gun owners on firearm policies: a three-stage nested study
Source: Inj Epidemiol. 2022 Oct 3;9:30. doi: 10.1186/s40621-022-00394-6 (PMC9527730; doi:10.1186/s40621-022-00394-6)
Supplement: Supplementary file 3 — Additional file 3. Appendix 3: Questions posed to the respondents. This appendix presents all the questions asked to the survey respondents. This appendix shows the results of the statistical analyses. [file 40621_2022_394_MOESM3_ESM.docx]

Appendix 3. Questions posed to the respondents

**Policy questions**

Based on the above message, please indicate your level of support or opposition to the following policies on a scale from 0 (fully oppose) to 10 (fully support):

1. Prohibiting a person subject to a temporary domestic violence restraining order from having a gun for the duration of the order

2. Background checks for private sales and at gun shows (universal background checks)

3. Prohibiting gun possession by people deemed to be a risk to themselves or others

4. Giving law enforcement officers discretion in whether or not to approve a concealed carry permit application

5. Prohibiting a person convicted of any violent crime from having a gun

6. Allowing people to shoot as a first option if they feel threatened, without any duty to retreat or to try to calm the situation (stand your ground laws)

**Engagement questions**

1. Based on the above message, would you contact a public official to express your support for a legislation designed to reduce gun violence?

2. Based on the above message, would you consider making a donation to an organization whose goal is to reduce gun violence?

3. Based on the above message, would you talk to your family members or friends about gun violence prevention?

4. Based on the above message, would you be willing to attend a meeting of public health advocates discussing policy proposals to reduce gun violence?

5. Based on the above message, would you be willing to testify at a public hearing in favor of legislation to reduce gun violence?

6. Based on the above message, would you be willing to write a letter to the editor supporting a policy to reduce gun violence?

7. Based on the above message, would you be willing to write a comment on an internet discussion that supports a policy to reduce gun violence?

8. Based on the above message, would you be willing to talk to other gun owners to try to gain their support for a policy to reduce gun violence?

9. Based on the above message, would you be willing to talk to non-gun owners to try to gain their support for a policy to reduce gun violence?
