# Supplementary material for: The effectiveness of value-based messages to engage gun owners on firearm policies: a three-stage nested study
Source: Inj Epidemiol. 2022 Oct 3;9:30. doi: 10.1186/s40621-022-00394-6 (PMC9527730; doi:10.1186/s40621-022-00394-6)
Supplement: Supplementary file 4 — Additional file 4. Appendix 4: Results of the first and second experiment for each engagement question. This appendix shows the results of the statistical analyses to measure the effect of the messages in experiments 1 and 2 on respondents’ willingness to engage in gun violence prevention in each of nine particular ways. [file 40621_2022_394_MOESM4_ESM.docx]

Appendix 4. Results of the first and second experiment for each engagement question

| Dependent Variable | Independent Variable | Coef. | Stand. Er. | P(>\|t\|) |
| --- | --- | --- | --- | --- |
| Experiment 1 | | | | |
| Contacting a public official | 1A. Belonging | -0.05 | 0.13 | 0.73 |
| Contacting a public official | 1B. Reciprocity | -0.09 | 0.13 | 0.49 |
| Contacting a public official | 1C. Freedom | -0.01 | 0.13 | 0.92 |
| Contacting a public official | 1D. Care | -0.25 | 0.13 | 0.07 |
| Contacting a public official | **1E. Loyalty** | **0.36** | **0.13** | **0.01** |
| Donating to a gun violence prevention organization | 1A. Belonging | 0.13 | 0.10 | 0.19 |
| Donating to a gun violence prevention organization | 1B. Reciprocity | 0.00 | 0.10 | 0.97 |
| Donating to a gun violence prevention organization | 1C. Freedom | -0.07 | 0.10 | 0.49 |
| Donating to a gun violence prevention organization | 1D. Care | -0.03 | 0.10 | 0.74 |
| Donating to a gun violence prevention organization | 1E. Loyalty | 0.14 | 0.10 | 0.17 |
| Talking to friends or family about prevention | **1A. Belonging** | **-0.40** | **0.14** | **0.01** |
| Talking to friends or family about prevention | **1B. Reciprocity** | **-0.43** | **0.14** | **0.00** |
| Talking to friends or family about prevention | **1C. Freedom** | **-0.38** | **0.15** | **0.01** |
| Talking to friends or family about prevention | **1D. Care** | **-0.42** | **0.14** | **0.00** |
| Talking to friends or family about prevention | 1E. Loyalty | 0.00 | 0.15 | 1.00 |
| Attending a meeting of public health advocates | 1A. Belonging | 0.14 | 0.11 | 0.21 |
| Attending a meeting of public health advocates | 1B. Reciprocity | -0.15 | 0.11 | 0.18 |
| Attending a meeting of public health advocates | 1C. Freedom | 0.01 | 0.11 | 0.91 |
| Attending a meeting of public health advocates | 1D. Care | 0.06 | 0.11 | 0.60 |
| Attending a meeting of public health advocates | 1E. Loyalty | 0.11 | 0.11 | 0.33 |
| Testifying at a public hearing in favor of a policy | **1A. Belonging** | **0.27** | **0.11** | **0.01** |
| Testifying at a public hearing in favor of a policy | 1B. Reciprocity | 0.11 | 0.11 | 0.29 |
| Testifying at a public hearing in favor of a policy | 1C. Freedom | 0.12 | 0.11 | 0.27 |
| Testifying at a public hearing in favor of a policy | 1D. Care | 0.19 | 0.11 | 0.08 |
| Testifying at a public hearing in favor of a policy | **1E. Loyalty** | **0.32** | **0.11** | **0.00** |
| Writing a letter to the editor in favor of a policy | **1A. Belonging** | **0.21** | **0.09** | **0.02** |
| Writing a letter to the editor in favor of a policy | 1B. Reciprocity | 0.07 | 0.09 | 0.43 |
| Writing a letter to the editor in favor of a policy | 1C. Freedom | 0.11 | 0.09 | 0.23 |
| Writing a letter to the editor in favor of a policy | 1D. Care | 0.13 | 0.09 | 0.14 |
| Writing a letter to the editor in favor of a policy | **1E. Loyalty** | **0.30** | **0.09** | **0.00** |
| Writing a comment online in favor of a policy | **1A. Belonging** | **0.29** | **0.10** | **0.00** |
| Writing a comment online in favor of a policy | 1B. Reciprocity | 0.12 | 0.10 | 0.22 |
| Writing a comment online in favor of a policy | 1C. Freedom | 0.10 | 0.10 | 0.29 |
| Writing a comment online in favor of a policy | 1D. Care | 0.00 | 0.10 | 0.97 |
| Writing a comment online in favor of a policy | **1E. Loyalty** | **0.27** | **0.10** | **0.00** |
| Gaining support from other gun owners | **1A. Belonging** | **0.45** | **0.11** | **0.00** |
| Gaining support from other gun owners | **1B. Reciprocity** | **0.33** | **0.11** | **0.00** |
| Gaining support from other gun owners | **1C. Freedom** | **0.38** | **0.12** | **0.00** |
| Gaining support from other gun owners | 1D. Care | 0.11 | 0.12 | 0.36 |
| Gaining support from other gun owners | **1E. Loyalty** | **0.76** | **0.12** | **0.00** |
| Gaining support from non-gun owners | **1A. Belonging** | **0.49** | **0.11** | **0.00** |
| Gaining support from non-gun owners | **1B. Reciprocity** | **0.42** | **0.11** | **0.00** |
| Gaining support from non-gun owners | **1C. Freedom** | **0.37** | **0.11** | **0.00** |
| Gaining support from non-gun owners | 1D. Care | 0.21 | 0.11 | 0.06 |
| Gaining support from non-gun owners | **1E. Loyalty** | **0.73** | **0.11** | **0.00** |
| Experiment 2 | | | | |
| Contacting a public official | **2A. Protection** | **-0.47** | **0.11** | **0.00** |
| Contacting a public official | **2B. Protection** | **-0.22** | **0.10** | **0.04** |
| Contacting a public official | 2C. 2nd Amendment activists | -0.11 | 0.13 | 0.40 |
| Contacting a public official | **2D. Hunters** | **-0.34** | **0.17** | **0.05** |
| Contacting a public official | **2E. Recreational owners** | **-0.41** | **0.13** | **0.00** |
| Contacting a public official | 2F. Active owners | 0.04 | 0.15 | 0.79 |
| Donating to a gun violence prevention organization | 2A. Protection | -0.12 | 0.07 | 0.10 |
| Donating to a gun violence prevention organization | 2B. Protection | -0.10 | 0.07 | 0.17 |
| Donating to a gun violence prevention organization | 2C. 2nd Amendment activists | 0.12 | 0.09 | 0.17 |
| Donating to a gun violence prevention organization | 2D. Hunters | 0.15 | 0.12 | 0.23 |
| Donating to a gun violence prevention organization | 2E. Recreational owners | -0.07 | 0.09 | 0.47 |
| Donating to a gun violence prevention organization | 2F. Active owners | 0.17 | 0.11 | 0.12 |
| Talking to friends or family about prevention | **2A. Protection** | **-0.30** | **0.12** | **0.03** |
| Talking to friends or family about prevention | 2B. Protection | -0.23 | 0.12 | 0.08 |
| Talking to friends or family about prevention | 2C. 2nd Amendment activists | -0.06 | 0.15 | 0.69 |
| Talking to friends or family about prevention | 2D. Hunters | -0.10 | 0.19 | 0.60 |
| Talking to friends or family about prevention | **2E. Recreational owners** | **-0.43** | **0.15** | **0.01** |
| Talking to friends or family about prevention | 2F. Active owners | -0.17 | 0.17 | 0.32 |
| Attending a meeting of public health advocates | 2A. Protection | -0.09 | 0.08 | 0.26 |
| Attending a meeting of public health advocates | 2B. Protection | 0.00 | 0.08 | 0.99 |
| Attending a meeting of public health advocates | 2C. 2nd Amendment activists | 0.14 | 0.10 | 0.19 |
| Attending a meeting of public health advocates | 2D. Hunters | 0.01 | 0.14 | 0.92 |
| Attending a meeting of public health advocates | 2E. Recreational owners | -0.19 | 0.11 | 0.07 |
| Attending a meeting of public health advocates | 2F. Active owners | -0.08 | 0.12 | 0.50 |
| Testifying at a public hearing in favor of a policy | 2A. Protection | -0.05 | 0.08 | 0.49 |
| Testifying at a public hearing in favor of a policy | 2B. Protection | 0.01 | 0.08 | 0.92 |
| Testifying at a public hearing in favor of a policy | 2C. 2nd Amendment activists | 0.19 | 0.10 | 0.06 |
| Testifying at a public hearing in favor of a policy | **2D. Hunters** | **0.27** | **0.13** | **0.04** |
| Testifying at a public hearing in favor of a policy | 2E. Recreational owners | -0.04 | 0.10 | 0.69 |
| Testifying at a public hearing in favor of a policy | 2F. Active owners | 0.14 | 0.12 | 0.21 |
| Writing a letter to the editor in favor of a policy | 2A. Protection | -0.01 | 0.06 | 0.84 |
| Writing a letter to the editor in favor of a policy | 2B. Protection | -0.01 | 0.06 | 0.94 |
| Writing a letter to the editor in favor of a policy | 2C. 2nd Amendment activists | 0.16 | 0.08 | 0.06 |
| Writing a letter to the editor in favor of a policy | **2D. Hunters** | **0.23** | **0.11** | **0.04** |
| Writing a letter to the editor in favor of a policy | 2E. Recreational owners | 0.10 | 0.08 | 0.26 |
| Writing a letter to the editor in favor of a policy | 2F. Active owners | 0.08 | 0.10 | 0.42 |
| Writing a comment online in favor of a policy | 2A. Protection | 0.01 | 0.07 | 0.91 |
| Writing a comment online in favor of a policy | 2B. Protection | -0.04 | 0.07 | 0.53 |
| Writing a comment online in favor of a policy | 2C. 2nd Amendment activists | 0.10 | 0.09 | 0.27 |
| Writing a comment online in favor of a policy | 2D. Hunters | 0.15 | 0.12 | 0.22 |
| Writing a comment online in favor of a policy | 2E. Recreational owners | -0.01 | 0.09 | 0.88 |
| Writing a comment online in favor of a policy | 2F. Active owners | -0.05 | 0.11 | 0.65 |
| Gaining support from other gun owners | 2A. Protection | 0.07 | 0.10 | 0.45 |
| Gaining support from other gun owners | 2B. Protection | 0.00 | 0.09 | 1.00 |
| Gaining support from other gun owners | 2C. 2nd Amendment activists | 0.18 | 0.12 | 0.13 |
| Gaining support from other gun owners | 2D. Hunters | 0.21 | 0.15 | 0.16 |
| Gaining support from other gun owners | 2E. Recreational owners | -0.21 | 0.12 | 0.08 |
| Gaining support from other gun owners | **2F. Active owners** | **0.40** | **0.13** | **0.00** |
| Gaining support from non-gun owners | 2A. Protection | 0.04 | 0.09 | 0.62 |
| Gaining support from non-gun owners | 2B. Protection | 0.16 | 0.09 | 0.08 |
| Gaining support from non-gun owners | 2C. 2nd Amendment activists | 0.21 | 0.11 | 0.06 |
| Gaining support from non-gun owners | 2D. Hunters | 0.22 | 0.14 | 0.12 |
| Gaining support from non-gun owners | 2E. Recreational owners | -0.07 | 0.11 | 0.52 |
| Gaining support from non-gun owners | **2F. Active owners** | **0.44** | **0.12** | **0.00** |
